# Construction of an evaluation system for medical staff’s occupational protection ability based on knowledge, attitude, and practice theory, and a cross-sectional survey of current conditions

**DOI:** 10.1186/s12912-023-01242-8

**Published:** 2023-03-22

**Authors:** Cunyue Zhao, Mingguang Yu, Aijun Wang, Xiaofen Xu, Xing Zheng

**Affiliations:** 1 Department of Spine Surgery, Shandong Electric Power Central Hospital, Jinan, China; 2 Department of Medical Oncology, Qilu Hospital, Cheeloo College of Medicine, Shandong University, Jinan, China; 3 Nursing Theory and Practice Innovation Research Center, Cheeloo College of Medicine, Shandong University, Jinan, China; 4 School of Nursing and Rehabilitation, Cheeloo College of Medicine, Shandong University, Jinan, China

**Keywords:** Medical staff, Occupational protection, Knowledge, Attitude, Practice, Cross-sectional survey

## Abstract

**Introduction:**

Medical personnel should be in close proximity and high frequency of contact with patients, and be exposed to physical, biological and chemical risk factors for a long time. The incidence of various occupational exposures is high. however, there is still a lack of the medical staff Occupational Protection Core Competence Evaluation Index system with high reliability and validity.

**Aim:**

Based on the theory of knowledge, attitude, and practice, the evaluation system of occupational protection ability of medical personnel was established, and the current situation of occupational protection ability of medical personnel at different levels was investigated, so as to take targeted training and intervention measures to improve the occupational protection ability of medical personnel and reduce the incidence of occupational exposure.

**Methods:**

Based on the knowledge, attitude, and practice theory, the index system of occupational protection core competence of medical personnel was initially constructed by literature retrieval, expert consultation, group discussion, semi-structured interview and other qualitative and quantitative methods, and the reliability and validity of the index system was tested by Delphi expert consultation method. By convenient cluster sampling method, from March to September 2021, the current status of occupational protection core competence of medical personnel was investigated among medical staff from one Class III Grade A hospital and two medical schools in Jinan City, Shandong Province, China.

**Results:**

The evaluation system for medical staff’s occupational protection ability included 3 first-level indexes, 11 second-level indexes, and 109 third-level indexes. A total of 684 valid questionnaires were collected from Grade III, Class A hospital medical staff and two medical school students in clinical practice in Shandong, China. Kruskal Walls test showed that there were significant differences in the overall distribution of occupational protection knowledge, attitude, and practice among registered nurses, nursing students, registered physicians, and physician students (*H* = 70.252, *P* < 0.001; *H* = 76.507, *P* < 0.001; *H* = 80.782, *P* < 0.001); there were statistical significance in the knowledge/ attitude/ practice of nursing and physician students at different levels (*H* = 33.733, *P* < 0.001; *H* = 29.158, *P* < 0.001; *H* = 28.740, *P* < 0.001).

**Conclusion:**

The results of the evaluation system for the medical staff’s occupational protection ability are reliable and can provide a reference for training the medical staff’s occupational protection ability. Managers should strengthen the training of theoretical knowledge of occupational protection ability of medical staff.

**Supplementary Information:**

The online version contains supplementary material available at 10.1186/s12912-023-01242-8.

## Introduction

Occupational exposure refers to long-term exposure to risk factors due to occupational relations, which may cause harm to the health and even the life of the practitioners [1]. The medical staff has to be exposed to physical, biological, chemical, and other risk factors for a long time because of close and high-frequency contact with patients. A survey of 206,711 health workers revealed that 63.0% of the respondents had experienced needle stick injuries and 73.4% had experienced biological occupational exposures [2]. Moreover, medical staff is often in a state of high stress in psychology and ergonomics because of work pressure and overwork [3]. A survey of 26,979 nurses in Taiwan showed that 13,392 (49.6%) nurses had experienced workplace violence, and there was a high rate of underreporting. According to a survey in China in 2018 by Yi et al. [4], 65.88% of participants were exposed to blood/body fluids thrice, and 31.2% experienced 1 ~ 5 occupational exposure in the past month. However, only 14.6% of participants submitted a blood/body fluid exposure report to a supervisor/official after every incident. With the normalization of epidemic prevention and control, the occupational protection of medical staff is particularly important. Nursing and physician students are more likely to be occupationally exposed due to their lack of technical skills, clinical experience, and familiarity with the environment. Occupational exposure will not only affect the physical health of the staff, but also cause greater psychological stress, which should be paid more attention to by educational administrators [5]. The investigation shows that strengthening education on clinical occupational protection is the main measure to reduce occupational exposure [6]. The knowledge of occupational protection has been included in the curriculum system abroad, but the related knowledge of occupational protection is very limited at present, and most nursing and physician students do not have clinical practice. It is limited to the improvement of attitude and practice only through classroom teaching. Training in post-clinical occupational protection is the most effective way to reduce occupational exposure to nursing and physician students. Although the occupational protection of medical staff has attracted the attention of researchers, there are some questionnaires about the knowledge, attitude, and practice of medical staff regarding occupational exposure to HIV [7] and hepatitis B [8], etc. The survey showed that only 12.1% of them had adequate knowledge of post-exposure to prophylactic treatment against HBV[8]. At present, there was a lack of a systematic evaluation index system for medical staff’s core competence in occupational protection, and most of the research tools on occupational protection were self-designed questionnaires. Their reliability and validity are poor [9, 10]. The teaching and training of occupational protection are scattered, and the construction and implementation of the training plan lack complete theoretical basis and systematic [11].

Therefore, This study uses a combination of qualitative and quantitative methods such as literature search, Delphi expert consultation, group discussion, and semi-structured interviews to construct an evaluation system for the core competencies of occupational protection for medical staff, Based on the theory of knowledge, attitude, and practice (KAP) theory. Meanwhile, The current situation of occupational protection knowledge, belief and attitude of registered medical personnel (registered doctors and registered nurses) and medical students (physician students and nursing students) was compared and analyzed, so as to provide certain theoretical basis for educational administrators to develop targeted measures of occupational protection training for medical personnel and improve the occupational protection ability of medical personnel. Thus, the incidence of occupational exposure of medical personnel can be reduced and the physical and mental health of medical personnel can be promoted.

## Methods

### Construction of the evaluation system of medical staff’s occupational protection ability based on the KAP theory

Literature search and evaluation: This study used “Knowledge, attitude, practice, medical staff, physician, nurse, nursing student, physician assistants, occupation protection, occupation expose, evaluation system” as the theme words and free words for literature retrieval, and a total of 4,081 till December,2020 published in PubMed, EMBASE, Cochrane Library, CNKI, and Wanfang were retrieved. 471 articles were reserved according to the literature screening process. According to the literature quality and the degree of correlation, 75 articles were reserved.

Literature evaluation: this study evaluated according to the hierarchy of evidence quality policy of Johns Hopkins nursing evidence-based practice (JHNEBP) [12]. The quality of 5 articles was still C grade, so they were deleted, and 70 articles were finally included in this study.

Based on the theory of KAP, preliminary construction of the core competency index system of medical staff’s occupational protection was completed through literature review, expert consultation, and qualitative interviews. The questionnaire consists of three parts: (1) letters to experts: mainly introducing the background, methods, significance, and points for attention; (2) a questionnaire of the core competency index system of medical staff’s occupational protection: experts evaluated the importance of all indexes according to Likert 5-grade scoring method (1 = very unimportant, 5 = very important); (3) the questionnaire of expert demography data (sex, age, professional title, educational background, etc.), the degree of familiarity with the contents of the questionnaire, the self-evaluation form of judgment basis.

Selection criteria: (1) bachelor degree or above; (2) senior associate degree or above; (3) working experience: 5 years or above teaching or management experience and 10 years or above professional experience; (4) scientific research ability: publishing 1 or more articles in core journals; (5) voluntary participation in this study.

This research adopts the critical value method to revise or delete the items according to the full score rate, the arithmetic mean, and the coefficient of variation [13]. If the above three items do not meet the threshold standard, then delete it; if 1 ~ 2 items do not meet the standard, then need the task group members given the expert’s opinion after argumentation, and then decide to revise or delete the indicators.

### Investigation on occupational protection ability of medical staff

#### Sample

The purpose of this study was to investigate the knowledge/attitude/ practice of occupational protection among medical staff and medical students in one ClassIII Grade A hospital and two medical schools in Shandong province from March to September 2021, using a convenient sampling. Inclusion criteria: (1) registered medical personnel or nursing and physician students in clinical practice; (2) consent to participate in the study. Exclusion Criteria: (1) Advanced medical staff; (2)Medical staff are being rotated at the moment.

#### Survey design and measures

This research adopts the self-made core competence evaluation system of medical personnel, which includes 3 first-level indexes, 11 second-level indexes, and 109 third-level indexes. It includes three dimensions of occupational protection knowledge, attitude, and practice. The higher the score, the stronger the ability for occupational protection.

A total of 684 valid questionnaires were collected among medical staff from a Grade III, Class A hospital and students in clinical practice from two medical schools in Shandong province. Among them, there were 162 registered nurses, 187 nursing students (64 undergraduates, 123 specialists), 70 registered physician and 265 physician students (221 undergraduates, 44 specialists).

### Data analysis

Epidata was used for data entry in this study. SPSS 25.0 was used for statistical analysis of the data, and the measurement data in line with normal distribution were used for descriptive analysis of mean ± standard deviation, and a t-test was used for comparison of differences between groups. For measurement data that do not conform to normal distribution. The median (P25, P75) was used for descriptive analysis, and the rank-sum test was used to compare the differences between groups. Frequency (percentage) was used for descriptive analysis, and the chi-square test was used to compare the differences between groups.

### Ethics

The study was reviewed by the Ethics Committee of Shandong University Qilu Hospital, ethics review number KYLL−202011−137−1. All methods of the study were carried out in accordance with the principles of the Helsinki Declaration. Written informed consent was obtained from all subjects before participating in the survey.

## Results

### Expert consultation results of evaluation system professional protection for medical staff

General information of experts: A total of 20 experts were consulted in the first round, and 19 of them gave replies, with an average age of (47.05 ± 6.99) years and an average working life of (25.68 ± 7.63) years. A total of 16 experts were consulted in the second round, and 15 experts gave replies, with an average age of (48.00 ± 5.64) years and an average working life of (30.20 ± 5.65) years (Table 1).

**Table 1:** Two rounds of expert general demographic data

Item	First round		Second round	
	*n*	Ratio(%)	*n*	Ratio(%)
Sex				
male	1	5.26	1	6.67
female	18	94.74	14	93.33
Age(year)				
30 ~ 40	3	15.79	1	6.67
40 ~ 50	8	42.11	5	33.33
>50	8	42.11	9	60.00
Working(year)				
<20	3	15.79	1	6.67
20 ~ 30	10	52.63	7	46.67
>30	6	31.58	7	46.67
Professional title				
Intermediate	3	15.79	1	6.67
senior vice	13	68.42	10	66.67
Senior	3	15.79	4	16.67
Highest education				
undergraduate	3	15.79	1	6.67
master	14	73.68	11	73.33
doctor	2	10.53	3	0.200

The active degree of experts: in the first round, among the 20 experts consulted by letter, 19 (95.00%) experts gave reply letters, and 12 (60%) experts put forward modification opinions; in the second round, among the 16 experts consulted by letter, 15 (93.75%) experts gave reply letters, 7 (46.67%) experts gave modification opinions.

The degree of expert authority is measured by the arithmetic average of the judgment coefficient and the degree of familiarity. In this study, the judgment coefficient of two rounds of experts were 0.900 and 0.934, respectively. The degree of familiarity were 0.884 and 0.907, respectively. The degree of expert authority were 0.892 and 0.921, respectively.

Coordination degree of experts: The coordination degree of expert opinions is generally represented by the Kendall W coordination coefficient and the coefficient of variation. In this study, the Kendall W coordination coefficient of the two rounds of expert opinions were 0.377 and 0.456, respectively (*P* < 0.05). The coefficients of variation of 10 s-level indicators in the first round were between 0.000 and 0.108, and the coefficients of variation of 11 s-level indicators in the second round were between 0.000 and 0.097. The coefficient of variation of 109 third-level indicators in the second round ranged from 0.000 to 0.160, indicating that the opinions of all experts tended to be consistent (Table 2).

**Table 2:** Results of expert letter consultation of second indicators

First Indicators	Second Indicators	Score of Importance		Coefficient of Variation	
		First round	Second round	First round	Second round
Knowledge	Basic knowledge of occupational protection	4.79 ± 0.42	4.93 ± 0.26	0.088	0.053
	Pathways related to occupational exposure	4.63 ± 0.50	4.87 ± 0.35	0.108	0.072
	Health effects of occupational exposure	4.68 ± 0.48	4.73 ± 0.46	0.103	0.097
	Basic protective measures against occupational exposure	5.00 ± 0.00	4.93 ± 0.26	0.000	0.072
	Post-occupational exposure management	5.00 ± 0.00	5.00 ± 0.00	0.000	0.000
Attitude	Severity of occupational exposure	4.63 ± 0.50	4.80 ± 0.41	0.108	0.085
	Importance of Occupational protection	4.89 ± 0.32	4.93 ± 0.26	0.654	0.072
Practice	Strictly follow the operation safety procedures	5.00 ± 0.00	5.00 ± 0.00	0.000	0.000
	Proper disposal of clinical waste	4.89 ± 0.32	4.87 ± 0.35	0.654	0.072
	Perform proper disinfection and isolation	——	4.80 ± 0.41	——	0.085
	Proper post-exposure treatment of occupational exposures	5.00 ± 0.00	4.87 ± 0.35	0.000	0.072

Concentration degree of experts: The concentration degree of expert opinions in this study is represented by the mean of importance score, coefficient of variation, and full score rate. After two rounds of expert consultation, the importance score of 109 three-level indicators was 4.27 ~ 5.00, the standard deviation was 0.00 ~ 0.74, and the full mark rate was 74.85%. According to the index screening method, the members of the research group summarized, analyzed, and sorted out the expert opinions that did not reach the cut-off value, and finally formed the evaluation index system of nurses’ occupational protection ability, including 3 first-level indicators, 11 second-level indicators and 109 third-level indicators (Supplementary Table 1).

### Current status of occupational protection of medical staff

#### Current status of occupational protection knowledge, attitude, and practice of medical staff

The median occupational protection knowledge/attitude/practice were 3.85(3.02, 4.69), 4.00 (3.14, 5.00), and 4.19 (3.26, 4.95) of medical staff. The median occupational protection knowledge was the smallest. Kruskal Walls test showed that there were statistical differences in the overall distribution of professional knowledge, attitude, and practice of medical staff (*H* = 31.761, *P* < 0.001) (Table 3). Bonferroni analysis of multiple comparisons through the multivariate rank-sum tests showed that there were statistically significant differences in the overall distribution of occupational protection knowledge and attitude, occupational protection knowledge and practice of medical staff (*P* < 0.001), but there was no statistically significant difference in occupational protection attitude and practice of medical staff (*P* > 0.05) (Table 4).

**Table 3:** occupational protection knowledge, attitude, and practice of medical staff (n = 684)

Item	M (P25, P75)	Kruskal Walls	
		*H*	*P*
Knowledge	3.85 (3.02, 4.69)	31.761	< 0.001
Attitude	4.00 (3.14, 5.00)		
Practice	4.19 (3.26, 4.95)		

**Table 4:** Multiple comparison of occupational protection knowledge, attitude, and practice of medical staff

Sample-1- Sample-2	Test Statistics	Std.Error	Std.Test Statistics	Sig.	Adj.Sig.
1–2	138.105	31.846	4.337	0.000	0.000
1–3	-168.316	31.846	-5.285	0.000	0.000
2–3	-30.211	31.846	-0.949	0.343	1.000

Note: 1 = knowledge 2 = attitude 3 = practice

#### Comparative analysis of knowledge, attitude, and practice of occupational protection among different categories of medical staff

Registered nurses, nursing students, registered physicians, and physician students’ occupational protection knowledge/attitude/practice average rank respectively for 414.79/365.80/344.12/266.32, 413.50/368.64/408.98/263.09, and 422.12/358.93/414.71/263.16. The rank means, of all, registered nurses were the largest, and physician students were the smallest. Kruskal Walls test showed that there were statistical differences in the overall distribution of occupational protection knowledge, attitude, and practice in different categories of medical staff (*H* = 70.252, *P* < 0.001; *H* = 76.507, *P* < 0.001; *H* = 80.782, *P* < 0.001) (Table 5).

**Table 5:** Occupational protection knowledge, attitude, and practice among different categories of medical staff

Item	Category	*n*	Rank	*H*	*P*
Knowledge	registered nurses	162	414.79	70.252	< 0.001
	nursing students	187	365.80		
	registered physicians	70	401.34		
	physician students	265	266.32		
Attitude	registered nurses	162	413.50	76.507	< 0.001
	nursing students	187	368.64		
	registered physicians	70	408.98		
	physician students	265	263.09		
Practice	registered nurses	162	422.12	80.782	< 0.001
	nursing students	187	358.93		
	registered physicians	70	414.71		
	physician students	265	263.16		

Bonferroni analysis showed that there were statistically significant differences in the overall distribution of occupational protection knowledge, attitude, and practice between physician students and registered nurses/nursing students/registered physicians after multiple rank-sum tests (*P* < 0.001). There were statistically significant differences in the overall distribution of occupational protection practice between nursing students and registered nurses (*P* = 0.016), but there were no statistically significant differences in the overall distribution of occupational protection knowledge, attitude, and practice between registered nurses and registered physicians (*P* > 0.05)(Table 6).

**Table 6:** Multiple comparison of occupational protection knowledge, attitude, and practice among different categories of medical staff

Item	Sample−1- Sample−2	Test Statistics	Std.Error	Std.Test Statistics	Sig.	Adj.Sig.
Knowledge	4−2	105.556	18.674	5.653	< 0.001	< 0.001
	4−3	145.890	26.276	5.552	< 0.001	< 0.001
	4−1	150.408	19.500	7.713	< 0.001	< 0.001
	2–3	−40.334	27.397	−1.472	0.141	0.846
	2−1	44.853	20.986	2.137	0.033	0.195
	3−1	4.518	27.967	0.162	0.872	1.000
Attitude	4−2	99.481	18.821	5.286	< 0.001	< 0.001
	4−3	135.022	26.483	5.098	< 0.001	< 0.001
	4−1	148.469	19.654	7.554	< 0.001	< 0.001
	2–3	−35.541	27.613	−1.287	−0.198	1.000
	2−1	48.988	21.152	2.316	0.021	0.123
	3−1	13.447	28.188	0.477	0.633	1.000
Practice	4−2	95.772	18.741	5.110	< 0.001	< 0.001
	4−3	151.549	26.370	5.747	< 0.001	< 0.001
	4−1	158.962	19.570	8.123	< 0.001	< 0.001
	2–3	−55.777	27.496	−2.029	0.043	0.255
	2−1	63.190	21.062	3.000	0.003	0.016
	3−1	7.413	28.068	0.264	0.792	1.000

Note: 1 = registered nurses 2 = nursing students 3 = registered physicians 4 = physician students

#### Comparative analysis of occupational protection knowledge among different categories of medical staff

The results of the Kruskal Walls Test showed that there were significant differences in the five dimensions of basic knowledge of occupational protection, occupational exposure-related pathways, occupational exposure to health, basic protective measures of occupational exposure, and post-exposure treatment among registered nurses, nursing students, registered physicians and physician students (*H* = 65.172, *P* < 0.001; *H* = 60.050, *P* < 0.001; *H* = 55.169, *P* < 0.001; *H* = 72.920, *P* < 0.001; *H* = 63.830, *P* < 0.001)(Table 7).

Bonferroni’s analysis showed that there were significant differences in the five dimensions of occupational protection knowledge between physician students and registered nurse/nursing students/ registered physicians (*P* < 0.001). There was no significant difference in the distribution of five dimensions of occupational protection among registered nurses, nursing students, and registered physicians (*P* > 0.05)(Supplementary Table 2).

#### Comparative analysis of occupational protection attitude among different categories of medical staff

Kruskal Walls test showed that there were significant differences in the overall distribution of occupational exposure severity and occupational exposure importance among registered nurses, nursing students, registered physicians, and physician students (*H* = 64.207, *P* < 0.001; *H* = 98.342, *P* < 0.001)(Table 7). Bonferroni analysis showed that there were significant differences in the two dimensions of occupational protection attitude between physician students and registered nurse/nursing students/registered physician (*P* < 0.001), there were no significant differences in the two dimensions among registered nurses, nursing students and registered physicians (*P* > 0.05)(Supplementary Table 3).

#### Comparative analysis of occupational protection practice among different categories of medical staff

Kruskal Walls test showed that there were statistically significant differences in the overall distribution of the four dimensions of registered nurses, nursing students, registered physicians, and physician students in occupational protection practice, including strict implementation of operational safety procedures, correct disposal of medical waste, correct disinfection and isolation, and correct post-exposure treatment (*H* = 87.230, *P* < 0.001; *H* = 79.016, *P* < 0.001; *H* = 82.345, *P* < 0.001; *H* = 72.845, *P* < 0.001) (Table 7). Bonferroni analysis showed that there were statistically significant differences in the overall distribution of the four dimensions of occupational protective practice between physician students and registered nurses/nursing students/registered physicians after multiple rank-sum tests (*P* < 0.001). There were statistically significant differences in the overall distribution of the two dimensions of strict implementation of the operation safety process and correct disinfection and isolation between nursing students and registered nurses (*P* = 0.003; *P* = 0.016). At the same time, there was a statistically significant difference in the overall distribution of the dimensions of correct occupational exposure post-treatment between nursing students and registered physicians (*P* = 0.015) (Supplementary Table 4).

**Table 7:** Dimensions of occupational protection knowledge, attitude, and practice among different categories of medical staff

Item	dimensionality	Category	*n*	Rank	*H*	*P*
Knowledge	Basic knowledge of occupational protection	registered nurses	162	410.69	65.172	< 0.001
		nursing students	187	370.13		
		registered physicians	70	389.57		
		physician students	265	268.88		
	Pathways related to occupational exposure	registered nurses	162	409.35	60.050	< 0.001
		nursing students	187	370.46		
		registered physicians	70	381.29		
		physician students	265	271.65		
	Health effects of occupational exposure	registered nurses	162	409.39	55.169	< 0.001
		nursing students	187	365.54		
		registered physicians	70	380.44		
		physician students	265	275.33		
	Basic protective measures against occupational exposure	registered nurses	162	415.01	72.920	< 0.001
		nursing students	187	366.88		
		registered physicians	70	402.76		
		physician students	265	265.05		
	Post-occupational exposure management	registered nurses	162	412.64	63.830	< 0.001
		nursing students	187	358.09		
		registered physicians	70	406.59		
		physician students	265	271.69		
Attitude	Severity of occupational exposure	registered nurses	162	405.37	64.207	< 0.001
		nursing students	187	369.12		
		registered physicians	70	403.00		
		physician students	265	269.30		
	Importance of Occupational protection	registered nurses	162	422.88	98.342	< 0.001
		nursing students	187	368.97		
		registered physicians	70	417.45		
		physician students	265	254.89		
Practice	Strictly follow the operation safety procedures	registered nurses	162	432.68	87.230	< 0.001
		nursing students	187	360.18		
		registered physicians	70	394.55		
		physician students	265	261.14		
	• Proper disposal of clinical waste	registered nurses	162	413.72	79.016	< 0.001
		nursing students	187	364.88		
		registered physicians	70	415.68		
		physician students	265	263.84		
	Perform proper disinfection and isolation	registered nurses	162	421.69	82.345	< 0.001
		nursing students	187	359.71		
		registered physicians	70	410.98		
		physician students	265	263.85		
	Proper post-exposure treatment of occupational exposures	registered nurses	162	415.98	72.845	< 0.001
		nursing students	187	352.67		
		registered physicians	70	423.00		
		physician students	265	269.15		

#### Current status of occupational protection knowledge, attitude, and practice of different categories of students

Undergraduate nursing students, specialist nursing students, undergraduate physician students, and specialist physician students’ occupational protection knowledge/attitude/practice rank average 263.57/270.94/202.01/195.91, 260.55/267.72/214.95/196.00, and 262.18/267.14/199.89/198.85, respectively. The rank means, of all, specialist nursing students was the highest, and that of specialist physician students was the lowest. Kruskal Walls test showed that there were statistical differences in the overall distribution of practice protection knowledge, attitude, and practice in different categories of students (*H* = 33.733, *P* < 0.001; *H* = 29.158, *P* < 0.001; *H* = 28.740, *P* < 0.001) (Table 8).

**Table 8:** Occupational protection knowledge, attitude, and practice of different categories of nursing student and physician assistants

Item	Category	*n*	Rank	*H*	*P*
Knowledge	undergraduate nursing students	64	263.57	33.733	< 0.001
	specialist nursing students	123	270.94		
	undergraduate physician students	44	202.01		
	specialist physician students	221	195.91		
Attitude	undergraduate nursing students	64	260.55	29.158	< 0.001
	specialist nursing students	123	267.72		
	undergraduate physician students	44	214.95		
	specialist physician students	221	196.00		
Practice	undergraduate nursing students	64	262.18	28.740	< 0.001
	specialist nursing students	123	267.14		
	undergraduate physician students	44	199.89		
	specialist physician students	221	198.85		

Bonferroni analysis showed that there were statistically significant differences in the overall distribution of protection knowledge, attitude, and practice between specialist physician students and specialist nursing students (*P* < 0.001). There were statistically significant differences in the overall distribution of protection knowledge, attitude, and practice between specialist physician students and undergraduate nursing students (*P* = 0.001; *P* = 0.003; *P* = 0.004) At the same time, there were statistically significant differences in the overall distribution of occupational protection knowledge and practice between undergraduate physician students and specialist nursing students (*P* = 0.014; *P* = 0.019) (Table 9).

**Table 9:** Multiple comparison of occupational protection knowledge, attitude, and practice among different categories of nursing students and physician students

	Sample−1- Sample−2	Test Statistics	Std.Error	Std.Test Statistics	Sig.	Adj.Sig.
Knowledge	4−3	6.104	21.347	0.286	0.775	1.000
	4−1	67.663	18.356	3.686	< 0.001	0.001
	4−2	75.032	14.547	5.158	< 0.001	< 0.001
	3−1	61.559	25.324	2.431	0.015	0.090
	3−2	68.928	22.716	3.034	0.002	0.014
	1–2	−7.369	19.931	−0.370	0.712	1.000
Attitude	4−3	18.959	21.503	0.882	0.378	1.000
	4−1	64.559	18.490	3.492	< 0.001	0.003
	4−2	71.724	14.653	4.895	< 0.001	< 0.001
	3−1	45.600	25.509	1.788	0.074	0.443
	3−2	52.765	22.881	2.306	0.021	0.127
	1–2	−7.165	20.076	−0.357	0.721	1.000
Practice	4−3	1.038	21.440	0.048	0.961	1.000
	4−1	63.331	18.435	3.435	0.001	0.004
	4−2	68.290	14.610	4.674	< 0.001	< 0.001
	3−1	62.293	25.434	2.449	0.014	0.086
	3−2	67.252	22.814	2.948	0.003	0.019
	1–2	−4.959	20.017	−0.248	0.804	1.000

Note:1 = undergraduate nursing students 2 = specialist nursing students 3 = undergraduate physician students 4 = specialist physician students

## Discussion

Long-term exposure of medical staff to physical, biological, chemical, and other risk factors will cause great harm to their physical and mental health. An investigation has shown that long-term exposure to radiation and long-term night workers will cause significant changes in REDOX reactions and inflammatory markers in their bodies [14]. Therefore, it is particularly important to pay attention to the occupational health of medical staff, and necessary measures should be taken to reduce the incidence of occupational exposure.

### The evaluation system for medical staff’s occupational protection ability based on the KAP theory is scientific and practical

The reliability of Delphi expert consultation results is closely related to the authority coefficient of experts and the degree of coordination concentration of experts. It is generally believed that the authority coefficient of experts is > 0.7, the coordination coefficient is about 0.5, and the variation coefficient is less than 0.25, which means that the reliability of experts is high [13]. In this study, the authority coefficients of the two rounds of experts were 0.892 and 0.921, the coordination coefficients were 0.377 and 0.456 (*P* < 0.05), and the coefficient of variation was between 0.000 and 0.160. Although the scores of ergonomics indexes were low, and the coefficients of variation of the three indexes of “correct filling in the Registration Form of Occupational Exposure Treatment after needle stick injury” and “correct taking preventive drugs after needle stick injury” were large, but the indexes in the expert consultation results all met the requirements of the critical value. And the system already contains all the clinical departments that have a higher incidence of needle stab and blood-borne occupational exposure indices such as knowledge, attitude, and practice, but also contains the junior indicators, such as the emergency department of a high incidence of workplace violence occupational exposure [15]. ICU, emergency department, and ergonomics occupational exposures were higher in the operating room. Studies have shown that ergonomics and psychosocial occupational exposure scores are the highest among nurses in ICU, pre-hospital emergency care, and operating room [3, 16]. There is a high incidence of occupational exposure to antineoplastic drugs in the oncology department [17]. Therefore, this system is suitable for more departments to evaluate the protective ability of medical staff and provide a certain theoretical basis for managers to take targeted training measures.

### Strengthen the training of theoretical knowledge of occupational protection ability of medical staff

This study showed that there were statistically significant differences in the overall distribution of occupational protection knowledge and attitude, occupational protection knowledge, and practice among medical staff. Medical staff had a relatively strong protection attitude and protection practice, but relatively poor protection knowledge. The survey by Choi et al. [8] 2018 showed that only 23.4% of people could mention all the key elements of post-exposure management of the hepatitis B virus, and 12.1% of people had enough understanding of post-exposure prophylaxis. Aminde et al.[18] 73.7% of the participants had poor knowledge about post-exposure prophylaxis for HIV.Though many (83.8%) had heard about PEP, just 10 (12.5%) had received formal training on PEP for HIV. In 2021, Morishima et al. [19] put forward the idea that radiation safety education is needed for personnel involved in the cardiology department. LI et al. [20] conducted simulation training on the COVID−19 virus for pediatric nurses in 2020. Simulation training can not only improve nurses’ COVID−19 emergency response ability but also alleviate psychological anxiety. Choi et al. [8] conducted a cross-sectional survey in South Korea in 2018. Improving the theoretical knowledge of the Zika virus can increase awareness of Zika virus prevention, promote the adoption of standard protective behavior, and reduce the incidence of occupational exposure. Therefore, standardized occupational protection training for medical staff is the most effective measure to reduce the incidence of occupational exposure.

### Strengthen the training of occupational protection ability of medical students, especially the training of physician students

This study showed that there were statistically significant differences in the overall distribution of occupational protection knowledge, attitude, and practice between physician students and registered nurses/nursing students/registered physicians (*P* < 0.001), and there were statistically significant differences in the overall distribution of protection knowledge, attitude and practice between specialist physician students and undergraduate nursing students/ specialist nursing students. The incidence of occupational exposure is relatively high. Mainly related to the fact that training accepted by the physician students is less, and the opportunity to participate in clinical operation during clinical practice is relatively small, the main time for case writing, etc.; besides, the practice nurses can carry on the related clinical operation as soon as they enter the clinic, but it is related to the lack of clinical experience, the unskilled technical operation, the unfamiliar ward environment, the psychological tension and so on The occurrence of occupational exposure not only affects physical health but also causes great psychological stress. Therefore, it is very important to strengthen the training in the occupational protection practice of nursing students. In addition to strengthening the training of occupational protection ability of students, it is especially necessary to strengthen the training of relevant knowledge of students, especially those with a low education background. At present, special systematic protection training systems for blood-borne [11] and biological [21] have been implemented in different regions, and the results show that the incidence of occupational exposure can be significantly reduced. At the same time, there are many factors affecting occupational protection. For example, El Ghaziri et al. [22] investigated in 2019 and found that the incidence of occupational exposure varied greatly among medical staff of different genders. Lana et al. [23] investigated 2015 and showed that the emotional intelligence of nursing students was also closely related to their health risks, and targeted and individualized methods should be adopted in occupational protection training for medical interns. At the same time, it is necessary to pay attention to the differences in different types of occupational exposure risks among different departments. The focus training on occupational exposure with high incidence in this department is more likely to achieve better training results.

## Conclusion

The evaluation system for medical staff’s occupational protection ability based on knowledge, attitude, and practice theory is scientific and practical, which can provide a reference for training the medical staff’s occupational protection ability. Managers should strengthen the training of theoretical knowledge of occupational protection ability of medical staff, meanwhile, pay attention to the training of occupational protection ability of nursing and physician students, especially for physician students with relatively low education level.

### Limitation

This study has the following limitations: (1) When constructing the core competence system of occupational protection for medical personnel in this study, the experts selected were limited by certain geographical conditions and other objective conditions, and no cross-international expert consultation was carried out. Therefore, this evaluation system needs further cultural adjustment, so as to broaden its scope of use. (2) This study only conducted a survey of the current situation in some areas, and the scope of the survey needs to be further expanded; Moreover, this study did not conduct a longitudinal study on the results. In the future, longitudinal investigation and analysis can be conducted to implement effective intervention based on the current investigation results, so as to truly improve the occupational protection ability of medical personnel and reduce the incidence of occupational exposure.

## Electronic supplementary material

Below is the link to the electronic supplementary material.

Supplementary Material 1

## Data Availability

Data materials are real and available. Xing Zheng should be contacted if someone wants to request the data from this study.
