# Supplementary material for: Construction of an evaluation system for medical staff’s occupational protection ability based on knowledge, attitude, and practice theory, and a cross-sectional survey of current conditions
Source: BMC Nurs. 2023 Mar 22;22:78. doi: 10.1186/s12912-023-01242-8 (PMC10031986; doi:10.1186/s12912-023-01242-8)
Supplement: Supplementary file 1 — Supplementary Material 1 [file 12912_2023_1242_MOESM1_ESM.docx]

Table S1 Results of expert letter consultation of third indicators

| Third Indicators | Score of importance | Coefficient of variation | Full mark rate (%) |
| --- | --- | --- | --- |
| 1.1.1 Master the basics of standard prevention | 4.93±0.26 | 0.053 | 93.33 |
| 1.1.2 Master the basics of physical occupational exposure | 4.93±0.26 | 0.053 | 93.33 |
| 1.1.3 Master the basic knowledge of biological occupational exposure | 4.87±0.35 | 0.072 | 86.67 |
| 1.1.4 Master the basic knowledge of chemical occupational exposure | 4.87±0.35 | 0.072 | 86.67 |
| 1.1.5 Master the basic knowledge of psychosocial occupational exposure | 4.67±0.49 | 0.105 | 66.67 |
| 1.1.6 Master the basic knowledge of ergonomic occupational exposure | 4.40±0.51 | 0.116 | 40.00 |
| 1.2.1 Master the relevant pathways of needle stick injury exposure | 5.00±0.00 | 0.000 | 100.00 |
| 1.2.2 Master the relevant pathways of occupational exposure to ionizing radiation | 4.73±0.46 | 0.097 | 73.33 |
| 1.2.3 Master the related pathways of blood-borne occupational exposure | 4.93±0.26 | 0.053 | 93.33 |
| 1.2.4 Master the related pathways of biological occupational exposure such as body fluids and secretions | 4.93±0.26 | 0.053 | 93.33 |
| 1.2.5 Master the related pathways of occupational exposure to antineoplastic drugs | 4.93±0.26 | 0.053 | 93.33 |
| 1.2.6 Master the relevant pathways of occupational exposure to antibiotics | 4.87±0.35 | 0.072 | 86.67 |
| 1.2.7 Master the relevant routes of chemical occupational exposure such as disinfectants | 4.87±0.35 | 0.072 | 86.67 |
| 1.2.8 Master the relevant pathways of workplace violence exposure | 4.80±0.41 | 0.085 | 80.00 |
| 1.2.9Master the related pathways of psychosocial occupational exposure such as work stress | 4.67±0.49 | 0.105 | 66.67 |
| 1.2.10 Master the relevant pathways of ergonomic occupational exposure | 4.33±0.49 | 0.113 | 33.33 |
| 1.3.1 Master the influence of needle stick injuries on physical health | 5.00±0.00 | 0.000 | 100.00 |
| 1.3.2 Master the effects of ionizing radiation on health | 4.87±0.35 | 0.072 | 86.67 |
| 1.3.3 Master the effects of blood-borne occupational exposure on health | 4.93±0.26 | 0.053 | 93.33 |
| 1.3.4 Master the effects of biological occupational exposure such as body fluids and secretions on health | 4.93±0.26 | 0.053 | 93.33 |
| 1.3.5 Master the effects of occupational exposure to antineoplastic drugs on health | 4.93±0.26 | 0.053 | 93.33 |
| 1.3.6 Master the effects of occupational exposure to antibiotics on health | 4.87±0.35 | 0.072 | 86.67 |
| 1.3.7 Master the effects of chemical occupational exposure such as disinfectants on health | 4.73±0.46 | 0.097 | 73.33 |
| 1.3.8 Master the impact of workplace violence exposure on physical health | 4.67±0.49 | 0.105 | 66.67 |
| 1.3.9 Master the effects of psychosocial occupational exposure such as work stress on physical health | 4.60±0.51 | 0.111 | 60.00 |
| 1.3.10 Master the effects of ergonomic occupational exposure on physical health | 4.27±0.46 | 0.097 | 26.67 |
| 1.4.1 Master the indications and methods of  hand washing and hand disinfection | 5.00±0.00 | 0.000 | 100.00 |
| 1.4.2 Master the indications and wearing methods of protective equipment | 5.00±0.00 | 0.000 | 100.00 |
| 1.4.3 Master the basic protective measures for occupational exposure to needle stick injuries | 5.00±0.00 | 0.000 | 100.00 |
| 1.4.4 Master the basic protective measures for occupational exposure to ionizing radiation | 4.87±0.35 | 0.072 | 86.67 |
| 1.4.5 Master the basic protective measures for blood-borne occupational exposure | 5.00±0.00 | 0.000 | 100.00 |
| 1.4.6 Master the basic protective measures for biological occupational exposure such as body fluids and secretions | 5.00±0.00 | 0.000 | 100.00 |
| 1.4.7 Master the basic protective measures for occupational exposure to antineoplastic drugs | 4.87±0.35 | 0.072 | 86.67 |
| 1.4.8 Master the basic protective measures for occupational exposure to antibiotics | 4.87±0.35 | 0.072 | 86.67 |
| 1.4.9 Master the basic protective measures of chemical occupational exposure such as disinfectants | 4.73±0.46 | 0.097 | 73.33 |
| 1.4.10 Master the basic protective measures for workplace violence exposure | 4.67±0.49 | 0.105 | 66.67 |
| 1.4.11 Master the basic protective measures for psychosocial occupational exposure such as work stress | 4.60±0.51 | 0.111 | 60.00 |
| 1.4.12 Master the basic protective measures for ergonomic occupational exposure | 4.40±0.51 | 0.116 | 40.00 |
| 1.5.1 Master the treatment and reporting process after needle stick injuries | 5.00±0.00 | 0.000 | 100.00 |
| 1.5.2 Master the treatment process after ionizing radiation and other physical occupational exposure | 4.87±0.35 | 0.072 | 86.67 |
| 1.5.3 Master the treatment process after blood-borne occupational exposure | 4.93±0.26 | 0.053 | 93.33 |
| 1.5.4 Master the preventive medication after blood-borne occupational exposure | 4.93±0.26 | 0.053 | 93.33 |
| 1.5.5 Master the treatment process after biological occupational exposure such as liquid and secretion | 4.87±0.35 | 0.072 | 86.67 |
| 1.5.6 Master the process after occupational exposure to antineoplastic drugs | 4.87±0.35 | 0.072 | 86.67 |
| 1.5.7 Master the treatment process after chemical occupational exposure such as antibiotics and disinfectants | 4.87±0.35 | 0.072 | 86.67 |
| 1.5.8 Master the treatment process after exposure to workplace violence | 4.73±0.46 | 0.097 | 73.33 |
| 1.5.9 Master the treatment process after psychosocial occupational exposure such as work stress | 4.67±0.49 | 0.105 | 66.67 |
| 1.5.10 Master the treatment process after ergonomic occupational exposure | 4.47±0.52 | 0.116 | 46.67 |
| 1.5.11 Master the assessment, prevention and follow-up after occupational exposure | 4.80±0.41 | 0.085 | 80.00 |
| 2.1.1 Awareness of the seriousness of occupational exposure to needlestick injuries | 5.00±0.00 | 0.000 | 100.00 |
| 2.1.2 Awareness of the seriousness of occupational exposure to ionizing radiation | 5.00±0.00 | 0.000 | 100.00 |
| 2.1.3 Awareness of the seriousness of blood-borne occupational exposure | 5.00±0.00 | 0.000 | 100.00 |
| 2.1.4 Awareness of the seriousness of biological occupational exposures such as body fluids and excreta | 4.87±0.35 | 0.072 | 86.67 |
| 2.1.5 Awareness of the seriousness of occupational exposure to antineoplastic drugs | 4.87±0.35 | 0.072 | 86.67 |
| 2.1.6 Awareness of the seriousness of occupational exposure to chemicals such as antibiotics and disinfectants | 4.73±0.46 | 0.097 | 73.33 |
| 2.1.7 Awareness of the seriousness of exposure to workplace violence | 4.60±0.51 | 0.116 | 60.00 |
| 2.1.8 Awareness of the seriousness of psychosocial occupational exposures such as work stress | 4.60±0.51 | 0.116 | 60.00 |
| 2.1.9 Awareness of the seriousness of occupational exposures such as adverse ergonomic environments and conditions | 4.40±0.51 | 0.116 | 40.00 |
| 2.2.1 Awareness of the importance of using a variety of protective equipment | 4.93±0.26 | 0.053 | 93.33 |
| 2.2.2 Awareness of the importance of changing unsafe behaviors | 4.87±0.35 | 0.072 | 86,67 |
| 2.2.3 Awareness of the importance of occupational protection training | 4.87±0.35 | 0.072 | 86.67 |
| 2.2.4 Awareness of the importance of adopting standardized occupational protection behavior | 4.93±0.26 | 0.053 | 93.33 |
| 2.2.5 Awareness of the importance of psychological adjustment and regular work and rest | 4.73±0.46 | 0.097 | 73.33 |
| 3.1.1 Correct placement of sterile sharps | 4.67±0.61 | 0.131 | 86.67 |
| 3.1.2 Open the ampoule the right way | 4.60±0.60 | 0.130 | 80.00 |
| 3.1.3 Wear the correct protective equipment when exposed to ionizing radiation | 4.87±0.35 | 0.072 | 86.67 |
| 3.1.4 Wear the correct protective equipment when touching different patients | 4.87±0.35 | 0.072 | 86.67 |
| 3.1.5 Gloves should be worn when touching patients' blood, body fluids, excreta and machine contaminated items | 4.93±0.26 | 0.053 | 93.33 |
| 3.1.6 Gloves should be worn before touching the patient's mucous membrane and non-intact skin | 4.87±0.35 | 0.072 | 86.67 |
| 3.1.7 Gloves should be replaced in time when the patient touches both the clean part and the contaminated part | 4.87±0.35 | 0.072 | 86.67 |
| 3.1.8 Gloves should be changed between the two patients | 4.93±0.26 | 0.053 | 93.33 |
| 3.1.9 Patients with blood, body fluids, excreta, etc. may be splashes, should wear eye masks, sick wear protective clothing | 4.87±0.35 | 0.072 | 86.67 |
| 3.1.10 Perform proper hand hygiene after touching patient's blood and surrounding environment | 4.87±0.35 | 0.072 | 86.67 |
| 3.1.11 Correct hand hygiene was performed before and after direct contact with each patient and before and after contact with the patient's mucous membrane damaged skin | 4.93±0.26 | 0.053 | 93.33 |
| 3.1.12 Perform proper hand hygiene before handling medications | 4.87±0.35 | 0.072 | 86.67 |
| 3.1.13 Wear protective equipment correctly when configuring antineoplastic drugs | 4.93±0.26 | 0.053 | 93.33 |
| 3.1.14 Antineoplastic drugs were dispensed in a safe exhaust environment | 4.87±0.35 | 0.072 | 86.67 |
| 3.1.15 Wear protective equipment correctly when configuring chemotherapeutic drugs such as disinfectants | 4.87±0.35 | 0.072 | 86.67 |
| 3.1.16 All operations were carried out according to the principle of force saving | 4.60±0.51 | 0.116 | 66.67 |
| 3.2.1 Correct disposal of contaminated and injurious waste | 4.93±0.26 | 0.053 | 93.33 |
| 3.2.2. Correct disposal of items contaminated with blood, body fluids and excreta of patients | 4.93±0.26 | 0.053 | 93.33 |
| 3.2.3 Correct disposal of household waste from isolated infected or suspected infected patients | 4.73±0.46 | 0.097 | 73.33 |
| 3.2.4 Correct handling of biological specimens to ensure safe submission | 4.80±0.41 | 0.085 | 80.00 |
| 3.2.5 Correct treatment of pathological waste | 4.87±0.35 | 0.072 | 86.67 |
| 3.2.6 Correct disposal of pharmaceutical waste such as discarded general drugs (such as antibiotics) | 4.80±0.41 | 0.085 | 80.00 |
| 3.2.7 Correct disposal of cytotoxic drug waste such as antineoplastic drugs | 4.93±0.26 | 0.053 | 93.33 |
| 3.2.8 Correct disposal of chemical medical waste | 4.80±0.41 | 0.085 | 80.00 |
| 3.2.9 Correct use of medical waste recycling containers | 4.87±0.35 | 0.072 | 86.67 |
| 3.2.10 Correct classify and label medical waste | 4.93±0.26 | 0.053 | 93.33 |
| 3.3.1 Correct clean and disinfect or replace the countertops contaminated by patients' blood, liquids, secretions, etc | 4.87±0.35 | 0.072 | 86.67 |
| 3.3.2 Correct clean and disinfect or replace the clothes and clothes contaminated by blood, liquid, secretion and so on | 4.87±0.35 | 0.072 | 86.67 |
| 3.3.3 Correct clean and disinfect areas contaminated by anti-tumor drugs in time | 4.80±0.41 | 0.085 | 80.00 |
| 3.3.4 Correct disinfection measures should be taken for reused medical instruments and appliances | 4.87±0.35 | 0.072 | 86.67 |
| 3.3.5 Patients with various diseases were isolated according to regulations | 4.87±0.35 | 0.072 | 86.67 |
| 3.4.1 After occupational exposure to needle-stick injuries, the wound should be treated correctly and timely according to the correct process | 5.00±0.00 | 0.000 | 100.00 |
| 3.4.2 Fill in Occupational Exposure Treatment Registration Form correctly after needle stick injury | 4.63±0.74 | 0.160 | 73.33 |
| 3.4.3 Preventive medication should be taken correctly after needle stick injuries | 4.63±0.74 | 0.160 | 73.33 |
| 3.4.4 Correct treatment after occupational exposure to ionizing radiation | 4.73±0.46 | 0.097 | 73.33 |
| 3.4.5 Correct treatment of blood-borne occupational exposure pollutants | 4.87±0.35 | 0.072 | 86.67 |
| 3.4.6 Correct filling in the Occupational Exposure Treatment Registration Form after blood-borne occupational exposure | 4.87±0.35 | 0.072 | 86.67 |
| 3.4.7 Preventive medication should be taken correctly after blood-borne occupational exposure | 4.87±0.35 | 0.072 | 86.67 |
| 3.4.8 Correct treatment, reporting and prevention of biological occupational exposure such as body fluid and excreta | 4.80±0.41 | 0.085 | 80.00 |
| 3.4.9 Correct treatment of antineoplastic drugs on skin or eyes | 4.73±0.46 | 0.097 | 73.33 |
| 3.4.10 Correct treatment of antineoplastic drug spillover | 4.73±0.46 | 0.097 | 73.33 |
| 3.4.11 Correct treatment of biological occupational exposure such as antibiotics and disinfectants | 4.73±0.46 | 0.097 | 73.33 |
| 3.4.12 Correct treatment of occupational exposure to workplace violence | 4.67±0.49 | 0.105 | 66.67 |
| 3.4.13 Correct handling of occupational exposure to work stress | 4.67±0.49 | 0.105 | 66.67 |
| 3.4.14 Correct handling of occupational exposure to adverse ergonomic environment and conditions | 4.60±0.51 | 0.111 | 60.00 |
| 3.4.15 Occupational post-exposure assessment, supervision and follow-up were standardized | 4.53±0.16 | 0.035 | 73.33 |

Table S2

Multiple comparisons of occupational protection knowledge among different categories of medical staff

| Item | Sample-1-  Sample-2 | Test Statistics | Std.Error | Std.Test Statistics | Sig. | Adj.Sig. |
| --- | --- | --- | --- | --- | --- | --- |
| Basic knowledge of occupational protection | 4-2 | 101.246 | 18.657 | 5.427 | <0.001 | <0.001 |
|  | 4-3 | 120.687 | 26.252 | 4.597 | <0.001 | <0.001 |
|  | 4-1 | 141.800 | 19.483 | 7.278 | <0.001 | <0.001 |
|  | 2-3 | -19.440 | 27.372 | -0.710 | 0.478 | 1.000 |
|  | 2-1 | 40.554 | 20.968 | 1.934 | 0.053 | 0.319 |
|  | 3-1 | 21.114 | 27.942 | 0.756 | 0.450 | 1.000 |
| Pathways related to occupational exposure | 4-2 | 98.807 | 18.714 | 5.280 | <0.001 | <0.001 |
|  | 4-3 | 109.640 | 26.333 | 4.164 | <0.001 | <0.001 |
|  | 4-1 | 137.702 | 19.542 | 7.046 | <0.001 | <0.001 |
|  | 2-3 | -10.833 | 27.456 | -0.395 | -0.693 | 1.000 |
|  | 2-1 | 38.895 | 21.032 | 1.849 | 0.064 | 0.386 |
|  | 3-1 | 28.062 | 28.027 | 1.001 | 0.317 | 1.000 |
| Health effects of occupational exposure | 4-2 | 90.205 | 18.723 | 4.818 | <0.001 | <0.001 |
|  | 4-3 | 105.104 | 26.346 | 3.989 | <0.001 | <0.001 |
|  | 4-1 | 134.057 | 19.552 | 6.856 | <0.001 | <0.001 |
|  | 2-3 | -14.898 | 27.470 | -0.542 | 0.588 | 1.000 |
|  | 2-1 | 43.851 | 21.042 | 2.084 | 0.037 | 0.223 |
|  | 3-1 | 28.953 | 28.068 | 0.264 | 0.792 | 1.000 |
| Basic protective measures against occupational exposur | 4-2 | 101.824 | 18.737 | 5.435 | <0.001 | <0.001 |
|  | 4-3 | 137.704 | 26.364 | 5.223 | <0.001 | <0.001 |
|  | 4-1 | 149.960 | 19.566 | 7.664 | <0.001 | <0.001 |
|  | 2-3 | -35.880 | 27.489 | -1.305 | 0.192 | 1.000 |
|  | 2-1 | 48.135 | 21.057 | 2.286 | 0.022 | 0.134 |
|  | 3-1 | 12.255 | 28.061 | 0.436 | 0.662 | 1.000 |
| Post-occupational exposure management | 4-2 | 86.394 | 18.743 | 4.609 | <0.001 | <0.001 |
|  | 4-3 | 134.899 | 26.373 | 5.115 | <0.001 | <0.001 |
|  | 4-1 | 140.941 | 19.573 | 7.201 | <0.001 | <0.001 |
|  | 2-3 | -48.505 | 27.499 | -1.764 | 0.078 | 0.467 |
|  | 2-1 | 54.548 | 21.064 | 2.590 | 0.010 | 0.058 |
|  | 3-1 | 6.043 | 28.071 | 0.215 | 0.830 | 1.000 |

Note: 1=registered nurses 2=nursing students 3=registered physicians 4=physicians students

Table S3

Multiple comparisons of occupational protection attitude among different categories of medical staff

| Item | Sample-1-  Sample-2 | Test Statistics | Std.Error | Std.Test Statistics | Sig. | Adj.Sig. |
| --- | --- | --- | --- | --- | --- | --- |
| Severity of occupational exposure | 4-2 | 99.814 | 18.651 | 5.352 | <0.001 | <0.001 |
|  | 4-3 | 133.696 | 26.244 | 5.094 | <0.001 | <0.001 |
|  | 4-1 | 136.064 | 19.477 | 6.986 | <0.001 | <0.001 |
|  | 2-3 | -33.882 | 27.364 | -1.238 | 0.216 | 1.000 |
|  | 2-1 | 36.250 | 20.961 | 1.729 | 0.084 | 0.502 |
|  | 3-1 | 2.367 | 27.933 | 0.085 | 0.932 | 1.000 |
| Importance of Occupational protection | 4-2 | 114.084 | 18.286 | 6.239 | <0.001 | <0.001 |
|  | 4-3 | 162.563 | 25.730 | 6.318 | <0.001 | <0.001 |
|  | 4-1 | 167.990 | 19.095 | 8.798 | <0.001 | <0.001 |
|  | 2-3 | -48.479 | 26.827 | -1.807 | 0.071 | 0.424 |
|  | 2-1 | 53.906 | 20.550 | 2.623 | 0.009 | 0.052 |
|  | 3-1 | 5.427 | 27.385 | 0.198 | 0.843 | 1.000 |

Note: 1=registered nurses 2=nursing students 3=registered physicians 4=physicians students

Table S4

Multiple comparisons of occupational protection practice among different categories of medical staff

| Item | Sample-1-  Sample-2 | Test Statistics | Std.Error | Std.Test Statistics | Sig. | Adj.Sig. |
| --- | --- | --- | --- | --- | --- | --- |
| Strictly follow the operation safety procedures | 4-2 | 99.038 | 18.631 | 5.316 | <0.001 | <0.001 |
|  | 4-3 | 133.407 | 26.216 | 5.089 | <0.001 | <0.001 |
|  | 4-1 | 171.539 | 19.456 | 8.817 | <0.001 | <0.001 |
|  | 2-3 | -34.368 | 27.334 | -1.257 | 0.209 | 1.000 |
|  | 2-1 | 72.500 | 20.939 | 3.463 | 0.001 | 0.003 |
|  | 3-1 | 38.132 | 27.903 | 1.367 | 0.172 | 1.000 |
| Proper disposal of clinical waste | 4-2 | 101.040 | 18.390 | 5.494 | <0.001 | <0.001 |
|  | 4-3 | 149.880 | 19.204 | 7.804 | <0.001 | <0.001 |
|  | 4-1 | 151.839 | 25.877 | 5.868 | <0.001 | <0.001 |
|  | 2-3 | 48.839 | 20.668 | 2.363 | 0.018 | 0.109 |
|  | 2-1 | -50.799 | 26.981 | -1.883 | 0.060 | 0.358 |
|  | 3-1 | -1.959 | 27.543 | -0.071 | 0.943 | 1.000 |
| Perform proper disinfection and isolation | 4-2 | 95.854 | 18.346 | 5.225 | <0.001 | <0.001 |
|  | 4-3 | 157.840 | 19.158 | 8.239 | <0.001 | <0.001 |
|  | 4-1 | 157.840 | 19.158 | 8.239 | <0.001 | <0.001 |
|  | 2-3 | -51.270 | 26.917 | -1.905 | 0.057 | 0.341 |
|  | 2-1 | 61.986 | 20.619 | 3.006 | 0.003 | 0.016 |
|  | 3-1 | 10.716 | 27.477 | 0.390 | 0.697 | 1.000 |
| Proper post-exposure treatment of occupational exposures | 4-2 | 83.520 | 18.635 | 4.482 | <0.001 | <0.001 |
|  | 4-3 | 146.830 | 19.460 | 7.545 | <0.001 | <0.001 |
|  | 4-1 | 153.855 | 26.222 | 5.867 | <0.001 | <0.001 |
|  | 2-3 | 63.310 | 20.943 | 3.023 | 0.003 | 0.015 |
|  | 2-1 | -70.334 | 27.341 | -2.573 | 0.010 | 0.061 |
|  | 3-1 | -7.025 | 27.909 | -0.252 | 0.801 | 1.000 |

Note: 1=registered nurses 2=nursing students 3=registered physicians 4=physicians students
